# Pelvic bone CT: can tin-filtered ultra-low-dose CT and virtual radiographs be used as alternative for standard CT and digital radiographs?

**DOI:** 10.1007/s00330-021-07824-x

**Published:** 2021-03-12

**Authors:** Christoph Stern, Stefan Sommer, Christoph Germann, Julien Galley, Christian W. A. Pfirrmann, Benjamin Fritz, Reto Sutter

**Affiliations:** 1 Radiology, Balgrist University Hospital, Forchstrasse 340, 8008 Zurich, Switzerland; 2 Faculty of Medicine, University of Zurich, Zurich, Switzerland; 3 Siemens Healthcare AG, Zurich, Switzerland; 4 SCMI, Swiss Center for Musculoskeletal Imaging, Balgrist Campus, Zurich, Switzerland

**Keywords:** Computed tomography, X-ray, Pelvic bones, Tin, Radiation dosage

## Abstract

**Objectives:**

To compare ultra-low-dose CT (ULD-CT) of the osseous pelvis with tin filtration to standard clinical CT (CT), and to assess the quality of computed virtual pelvic radiographs (VRs).

**Methods:**

CT protocols were optimized in a phantom and three pelvic cadavers. Thirty prospectively included patients received both standard CT (automated tube voltage selection and current modulation) and tin-filtered ULD-CT of the pelvis (Sn140kV/50mAs). VRs of ULD-CT data were computed using an adapted cone beam–based projection algorithm and were compared to digital radiographs (DRs) of the pelvis. CT and DR dose parameters and quantitative and qualitative measures (1 = worst, 4 = best) were compared. CT and ULD-CT were assessed for osseous pathologies.

**Results:**

Dose reduction of ULD-CT was 84% compared to CT, with a median effective dose of 0.38 mSv (quartile 1–3: 0.37–0.4 mSv) versus 2.31 mSv (1.82–3.58 mSv; *p* < .001), respectively. Mean dose of DR was 0.37 mSv (± 0.14 mSv). The median signal-to-noise ratio (SNR) and contrast-to-noise ratio (CNR) of bone were significantly higher for CT (64.3 and 21.5, respectively) compared to ULD-CT (50.4 and 18.8; *p* ≤ .01), while ULD-CT was significantly more dose efficient (figure of merit (FOM) 927.6) than CT (FOM 167.6; *p* < .001). Both CT and ULD-CT were of good image quality with excellent depiction of anatomy, with a median score of 4 (4–4) for both methods (*p* = .1). Agreement was perfect between both methods regarding the prevalence of assessed osseous pathologies (*p* > .99). VRs were successfully calculated and were equivalent to DRs.

**Conclusion:**

Tin-filtered ULD-CT of the pelvis at a dose equivalent to standard radiographs is adequate for assessing bone anatomy and osseous pathologies and had a markedly superior dose efficiency than standard CT.

**Key Points:**

*• Ultra-low-dose pelvic CT with tin filtration (0.38 mSv) can be performed at a dose of digital radiographs (0.37 mSv), with a dose reduction of 84% compared to standard CT (2.31 mSv).*

*• Tin-filtered ultra-low-dose CT had lower SNR and CNR and higher image noise than standard CT, but showed clear depiction of anatomy and accurate detection of osseous pathologies.*

*• Virtual pelvic radiographs were successfully calculated from ultra-low-dose CT data and were equivalent to digital radiographs.*

**Supplementary Information:**

The online version contains supplementary material available at 10.1007/s00330-021-07824-x.

## Introduction

The radiation dose of a standard CT of the osseous pelvis is many times higher compared to pelvic radiographs [1] and carries a higher risk of developing cancer, especially in young adults [2]. In many orthopedic patients, the bone and joints of the pelvis are frequently evaluated by standard radiographs as first-line imaging, commonly with anteroposterior and cross-table axial views [3, 4]. As a projection-based technique, radiographs are susceptible to tilt and rotation errors or superposition by soft tissue or gas which can result in diagnostic difficulties. Hence, in several patients, an additional CT scan of the pelvis is performed to exclude fractures or as part of preoperative planning as it provides detailed cross-sectional anatomic information.

While techniques such as automatic tube voltage, tube current modulation, and iterative image reconstruction have resulted in a reduced radiation dose, CT still results in much higher radiation exposure for the patient than radiographs. A promising new CT technique is the use of an additional tin filter, which is placed in front of the X-ray tube for spectral shaping of the X-ray beam. Low-energy photons that contribute little to image quality of high-contrast structures such as bone but expose the patient to higher radiation are filtered out. This results in hardening of the X-ray spectrum with more penetrable photons and reduction of radiation dose [5, 6]. CT studies of the thorax [7–9] and abdomen [10] showed promising results with remarkable reduction of radiation dose by using this technique without compromise in image quality.

To our knowledge, no study has investigated the application of a tin filter for pelvic CT to lower the radiation dose to the level of pelvic radiographs. We hypothesize that it is feasible to acquire a tin-filtered ultra-low-dose CT (ULD-CT) of the osseous pelvis in diagnostic image quality with radiograph-equivalent dose and to calculate an additional diagnostic virtual pelvic radiograph of the CT data.

Therefore, the purpose of the study was to establish tin-filtered ULD-CT of the osseous pelvis at a dose equivalent to digital radiographs (DRs) of the pelvis and to compare the image quality and clinical utility to standard clinical CT (CT). The second purpose was to assess the quality of calculated virtual pelvic radiographs (VRs).

## Materials and methods

This single-center prospective study was approved by the cantonal ethics committee and is in accordance with the Declaration of Helsinki, the principles of Good Clinical Practice, and other Swiss regulations. Written informed consent was obtained from all participants and permission for scientific use of cadavers existed.

### Pelvic phantom and pelvic cadavers

Materials and methods for the examination of the pelvic phantom and pelvic cadavers are described in the supplementary data.

### Study participants

Patients were prospectively included after having received a standard dose pelvic CT at Balgrist University Hospital for clinical indications, followed by an additional ultra-low-dose CT of the pelvis with tin filtration for this study between April 2019 and January 2020. Pelvic DRs that were performed for clinical indications were included for analysis in the study. Inclusion criteria were adult patients who were referred for pelvic CT. Exclusion criteria comprised metal implants in the pelvis and proximal femur, tumor, or pregnancy.

### Imaging technique

The standard non-contrast CT of the osseous pelvis without tin filtration was part of the clinical routine imaging workup at Balgrist University Hospital either on a 64-slice CT scanner (SOMATOM Definition AS, Siemens Healthineers) or on a 128-slice CT scanner (SOMATOM Edge Plus, Siemens Healthineers). On both CT scanners, images were acquired with automated tube voltage selection (CARE kV, reference 120kV) and tube current modulation (CARE Dose4D, reference 147mAs), a collimation width of 0.6 mm, a rotation time of 0.5 s, and a pitch of 0.8.

After the standard CT, all study participants received a non-contrast ultra-low-dose pelvic CT with tin filtration (protocol Sn140kV/50mAs) of the same coverage in the z-axis on the 128-slice CT scanner (SOMATOM Edge Plus, Siemens Healthineers) either at Balgrist University Hospital or at the Swiss Center for Musculoskeletal Imaging. The ULD-CT protocol was developed in pelvic cadavers, which is shown in the supplementary material. Settings of the ULD-CT protocol were as follows: a fixed tube voltage of 140 kV with tin filtration (Sn), fixed tube current of 50mAs, collimation width of 0.6 mm, rotation time of 1 s, and a pitch of 0.8.

Both the standard CT and ULD-CT were acquired in supine position with 15° internal rotation of the legs.

#### CT image reconstruction

Both the standard CT and the tin-filtered ultra-low-dose pelvic CT were reconstructed in the axial (3 mm), coronal (2 mm), and sagittal (2 mm) image plane using a bone kernel (Br 57). Furthermore, for both CT scans, subsequent 3D volume rendering technique (VRT) reconstructions of the pelvis were calculated using axial image reconstructions (1.5 mm) in soft tissue kernel (Br 38). Advanced modeled iterative reconstruction (ADMIRE) strength level 3 was applied for all image reconstructions.

#### Computed virtual radiographs

Virtual radiographs of ULD-CT data were generated using an adapted 3D cone beam projection algorithm based on the implementation from Kyungsang [11] in MATLAB (The MathWorks, Inc., vers. R2018b). Axial image reconstructions of 0.5-mm thickness in bone kernel (Br 57) of the ULD-CT were used as input. To avoid projection errors, the VR was calculated according to the settings of a digital orthopedic pelvic radiograph with a virtual film-focus distance of 1.2 m and with the virtual center beam directed to the midpoint between a line connecting the anterosuperior iliac spines and the superior pubic symphysis. Internal rotation was fulfilled as ULD-CTs were acquired with legs 15° internally rotated [12].

#### Digital radiographs

A search of the picture archiving and communication system (PACS) of Balgrist University Hospital was performed for available digital anteroposterior and cross-table axial pelvic radiographs. Dose parameters were extracted from the dose report.

### CT and radiograph analysis

Two musculoskeletal radiologists (C.S. with 7 years of experience (reader 1) and C.G. with 6 years of experience (reader 2)) interpreted standard CT, ULD-CT, DR, and VR independently on a PACS workstation and were blinded to each other. Images were anonymized and displayed in random order. Both readers were blinded to clinical information and imaging results.

#### Quantitative image analysis

For standard CT and ULD-CT, scan length and dose parameters were extracted from the dose report: volume CT dose index (CTDI_vol_), dose length product (DLP), tube voltage (kV), and tube current-time product (mAs). Effective CT dose was estimated by multiplying the DLP with a standard conversion factor *k* for the adult pelvis of 0.013 mSv/mGy*cm [13]. Effective dose of DR was estimated by multiplying the dose area product with *k* = 0.00029 mSv/mGy*cm^2^ [14].

CT values (HU) of the muscle and cortical bone were measured by reader 1 on reconstructed axial 3-mm slices in bone kernel. For the muscle, regions of interest (ROIs) of equal size were placed in the left gluteal muscle, and for the cortical bone, ROIs were placed in the cortical bone of the proximal shaft of the left femur. Image noise was defined as the standard deviation of the CT attenuation in air, which was measured outside the body anterior to the pelvic wall with ROIs of equal size [10]. All ROIs were placed in nearly identical locations using anatomical landmarks.

Signal-to-noise ratio (SNR), contrast-to-noise ratio (CNR), and a figure of merit (FOM) to normalize the CNR to compare the dose efficiency between protocols were calculated for cortical bone for standard CT and ULD-CT, respectively. The following equations were used: SNR = (mean_cortical bone/SD_background air); CNR = (mean_cortical bone − mean muscle)/(SD_cortical bone); FOM = CNR^2^/effective dose [10].

#### Qualitative image analysis

For standard CT and ULD-CT, both readers rated the following parameters on a 4-point Likert scale: depiction of anatomy (1 = poor, 2 = fair, 3 = moderate, 4 = good), image noise (1 = very high, 2 = high, 3 = moderate, 4 = minimal), image artifacts (1 = very strong, 2 = strong, 3 = weak, 4 = none), and quality of 3D VRT reconstructions (1 = poor, 2 = fair, 3 = moderate, 4 = good). Supplementary table 1 shows definitions of all ratings.

For DR and VR, depiction of anatomy (1 = poor, 2 = fair, 3 = moderate, 4 = good) was rated (supplementary table 1).

#### Imaging findings and measurements

Both readers evaluated standard CT and ULD-CT for the presence or absence of fracture, osteoarthritis, cam deformity, and bone islands [15]. For each category, diagnostic confidence was rated on a 4-point Likert scale (1 = low, 2 = moderate, 3 = high, 4 = very high).

In order to evaluate whether the anatomical projection on the virtual radiographs is feasible, we determined two exemplary measures on the VRs for the right and the left hip joint (lateral center-edge (CE) angle and Sharp angle [16–18]). CE and Sharp angles of both hip joints were also measured on DRs.

### Statistical analysis

REDcap software (version 9.3.4, Vanderbilt University) was used to enter, store, and manage data with an electronic case report form for every study participant. In the “Results” section, all results of reader 1 and agreement between readers 1 and 2 were reported. General descriptive statistics were used and ordinal data was reported as median with 25th percentile (Q1) and 75th percentile (Q3), whereas continuous data as mean with standard deviation (SD). The Shapiro-Wilk test was applied to test for normal distribution.

CT dose parameters, CT values of muscle and bone, and SNR, CNR, and FOM were compared between the standard and ultra-low-dose protocol using the Wilcoxon signed-rank test. Furthermore, the Wilcoxon signed-rank test was used to evaluate differences in depiction of anatomy, image noise, image artifacts, and quality of 3D VRT reconstruction between standard CT und ULD-CT as well as to compare depiction of anatomy on DR and VR.

Prevalence of assessed osseous pathologies was calculated for standard CT und ULD-CT and the McNemar test was used for comparison. Diagnostic confidence of every category was compared with the Wilcoxon signed-rank test.

Bland-Altman plots [19] were used to compare radiographic angle measurements on DR and VR.

To measure interreader agreement, kappa statistics (*ĸ*) for ordinal and intraclass correlation coefficient (ICC) for continuous data were calculated. Effect size for *ĸ* was interpreted as slight (0–0.20), fair (0.21–0.40), moderate (0.41–0.60), substantial (0.61–0.80), or excellent (0.81–1.00) [20] and for ICC values > 0.75 were considered as good and > 0.9 as excellent agreement [21].

For statistical analysis, SPSS (version 23, IBM Corporation) was used. For any value of *p* < 0.05, significance was assumed.

## Results

### Pelvic phantom and pelvic cadavers

A pelvic phantom and three pelvic cadavers were included to establish and optimize CT protocols (see supplementary data).

### Study participants

Thirty patients were included (11 males, 19 females, mean age 38 years ± SD 16.7 years). The mean body mass index was 25.8 ± SD 5.2. The calculation of 3D VRT reconstructions (standard CT and ULD-CT) and of virtual radiographs was successful for all patients (30/30, 100%). Clinical digital radiographs were available in 28 of 30 patients (93.3%).

#### Effective dose and quantitative analysis

Effective dose of ULD-CT with median 0.38 mSv (Q1–Q3: 0.37 mSv–0.4 mSv) was significantly lower compared to standard CT with median 2.31 mSv (1.82 mSv–3.58 mSv) (*p* < .001), resulting in a 6.1-fold dose reduction (−83.5% dose reduction). Table 1 shows scan length and other dose parameters of standard CT and ULD-CT. In 24 of 28 digital pelvic radiographs (85.7%), the dose area product was available and the estimated effective dose was mean 0.37 mSv (± 0.14 mSv).

**Table 1:** Scan length and CT dose parameters of patient scans

	Standard CT^†^	Ultra-low-dose CT^††^	*p* value*
Tube current (kV)	90, 100, 110, 120, 140	Sn140	NA
Tube current-time product (mAs)	106–366	50	NA
CTDI_vol_ (mGy)	6.73 (5.24–9.97)	1.07	<. 001
DLP (mGy*cm)	177.85 (139.9–275.45)	28.85 (28.03–30.93)	<. 001
Scan length (mm)	267.5 (260.5–284.6)	266.4 (259.5–278.7)	.44
Effective dose (mSv)^†††^	2.31 (1.82–3.58)	0.38 (0.37–0.4)	<. 001

^†^CT parameters were automatically adapted to patient habitus

^††^A fixed protocol was used with fixed CT parameters (kV, mAs, CTDI_vol_)

^†††^Effective dose (mSv) was estimated by multiplying the DLP with a standard conversion factor *k* for the adult pelvis of 0.013 mSv/mGy*cm

Values are displayed as median with 25th percentile and 75th percentile in parentheses (Q1–Q3)

*p values calculated with the Wilcoxon signed-rank test

*CTDI*_*vol*_ volume CT dose index, *DLP* dose length product, *kV* kilo volt, *mAs* milliampere seconds, *mGy* milligray, *mSv* millisievert, *NA* not applicable, *Sn* tin filter

CT values of standard CT and ULD-CT were different for muscle with median 57.5 HU (52.0 HU–61.3 HU) and 54 HU (50.8 HU–57.3 HU) (*p* < .01) and for cortical bone with median 1497.5 HU (1411.8 HU–1663.0 HU) and 1124 HU (1101.8 HU–1174.8 HU) (*p* < .001), respectively. For standard CT, SNR with median 64.3 (50.5–73.4) and CNR with median 21.5 (16.5–29.2) were higher compared to ULD-CT with median SNR 50.4 (47.4–55.8) and median CNR 18.8 (16.7–22) (*p* < .01 and *p* = .01). However, ULD-CT was more dose efficient compared to standard CT with a FOM of median 927.6 (700.5–1316.2) versus median 167.6 (103.9–360) (*p* < .001).

#### Qualitative analysis

**Fig. 1:**
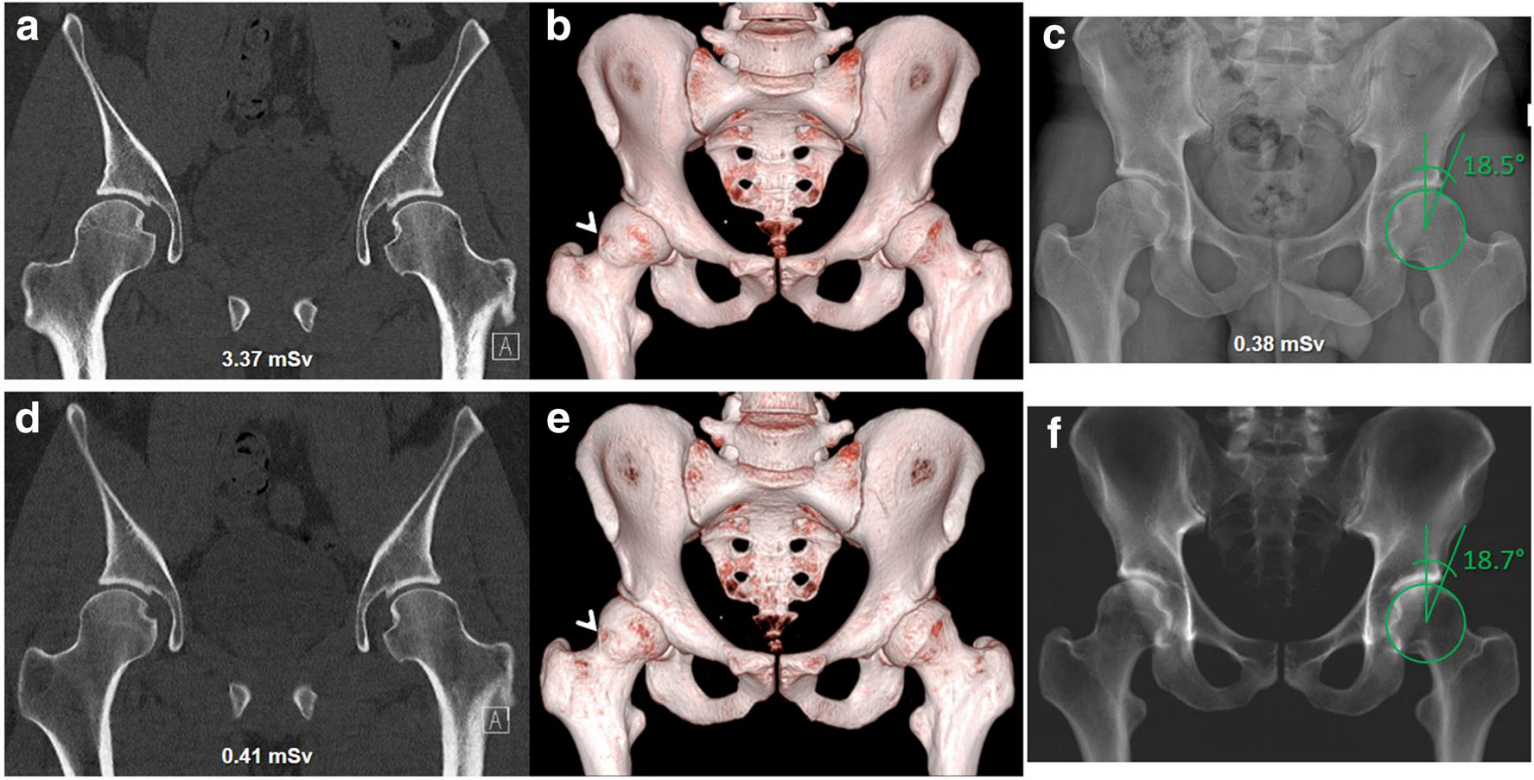
A 25-year-old male with symptomatic hip dysplasia of both sides. Reformatted coronal CT image of both hip joints, scanned with the standard protocol (**a**) and ultra-low-dose protocol with tin filtration (Sn140kV/50mAs; **d**) show clear depiction of anatomy. 3D VRT pelvic reconstruction of the standard CT (**b**) and ultra-low-dose CT (**e**) both demonstrate clear pelvic anatomy. Calculated virtual pelvic a.p. radiograph of the ultra-low-dose CT (**f**) shows clear depiction of anatomy compared to the digital a.p. radiograph of the pelvis (**c**). Measurement of the left center-edge angle was almost identical with 18.7° for the virtual and 18.5° for the digital radiograph. Note cam deformity of the right femur in **b** and **e** (arrowheads)

Both standard CT and ULD-CT were of good image quality: depiction of anatomy was median 4 (4–4) and 4 (4–4) (*p* = .1) and quality of 3D VRT reconstructions was median 4 (3–4) and 4 (3–4) (*p* = .06) (Fig. 1). Image noise was lower for standard CT with median 4 (4–4) compared to ULD-CT with median 3 (3–4) (*p* < .001). However, ULD-CT showed less image artifacts with median 4 (3.75–4) compared to standard CT with median 3.5 (3–4) (*p* = .03) (supplementary Fig. 2).

DR and VR both showed good depiction of anatomy with median 4 (4–4) and 4 (4–4), respectively (*p* = .08) (Fig. 1). Rate of disagreement for readers 1 and 2 ranged between 0 and 23.3% and did not exceed 1 Likert point (supplementary table 2).

#### CT imaging findings

**Fig. 2:**
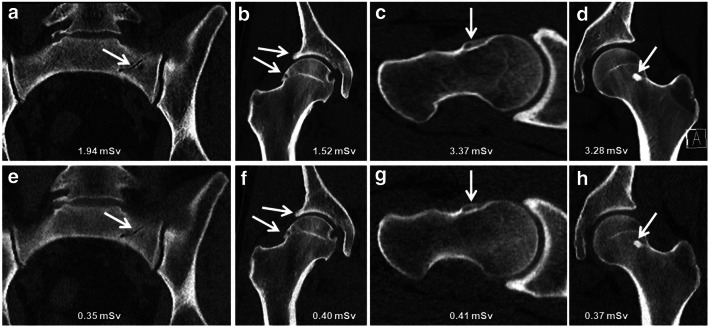
Standard CT (**a**–**d**) and corresponding ultra-low-dose CT (Sn140kV/50mAs) with tin filtration (**e**–**h**) of the pelvis in 4 different patients. A 55-year-old male (**a** and **e**) with a non-displaced fracture of the left sacrum (arrow). A 38-year-old male (**b** and **f**) with osteoarthritis of the right hip joint (arrows). A 25-year-old male (**c** and **g**) with cam configuration of the right anterior femoral head/neck junction (arrow). A 30-year-old female (**d** and **h**) with bone island in the left femoral head/neck junction

Standard CT and ULD-CT showed perfect agreement regarding the presence or absence of assessed osseous pathologies: 3 of 30 (10%; 95% confidence interval (CI): 2.9%, 24.3%) had fracture, 14 of 30 (46.7%; 95% CI: 29.8%, 64.1%) had osteoarthritis, 11 of 30 (36.7%; 95% CI: 21.3%, 54.5%) had cam configuration, and 23 of 30 (76.7%; 95% CI: 59.6%, 88.9%) had bone island (all *p* > .99) (Fig. 2). Interreader agreement between the two musculoskeletal radiologists was excellent for both standard CT (*ĸ* = .9) and ULD-CT (*ĸ* = .85). Diagnostic confidence to detect fracture, osteoarthritis, cam configuration, and bone island was very high for readers 1 and 2, and for both readers, no difference was observed between standard CT and ULD-CT (all 4 (4–4): *p* value reader 1: .1–.56).

#### Virtual radiographs: Measurements

**Fig. 3:**
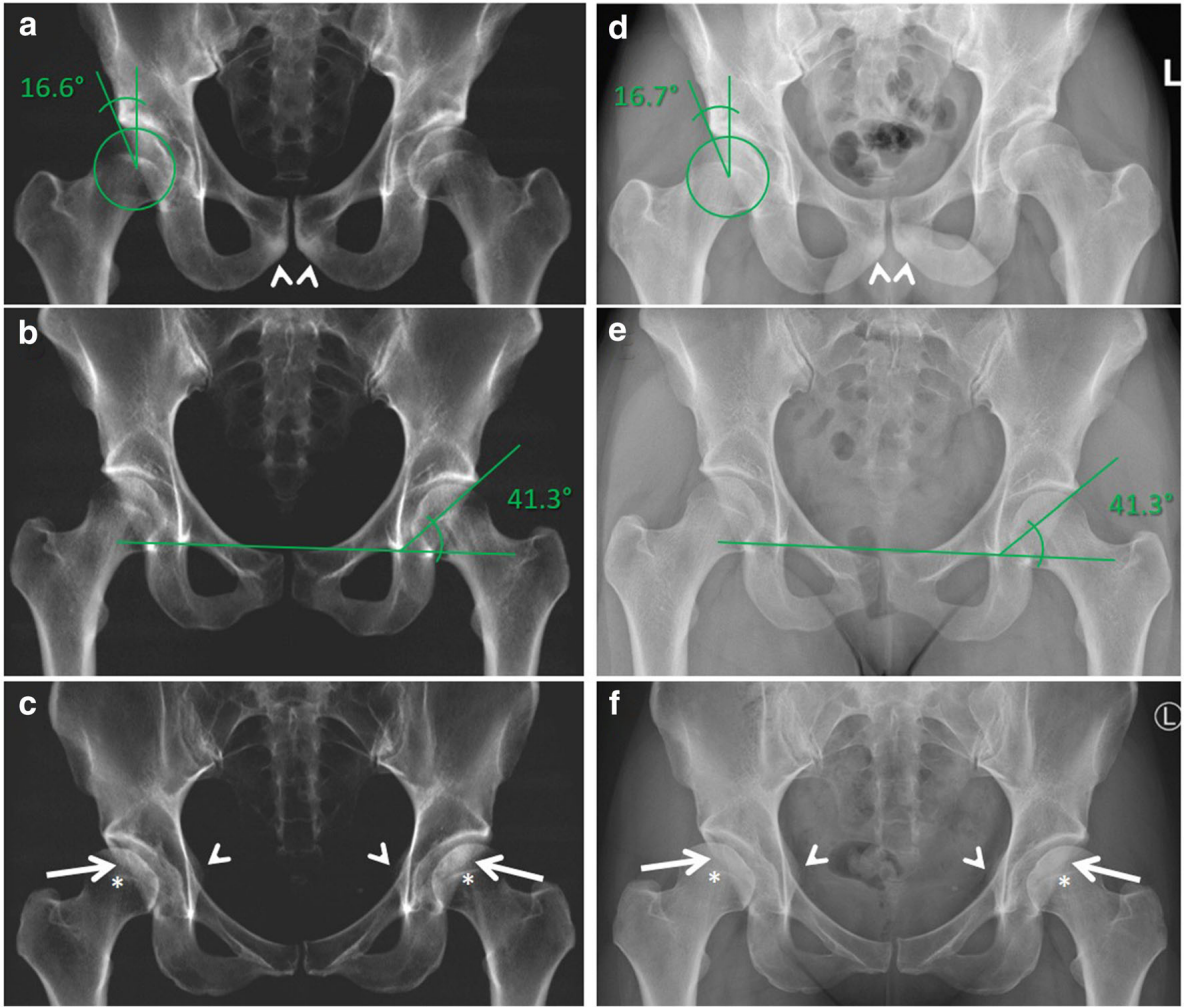
Calculated virtual a.p. pelvic radiographs of the ultra-low-dose CT (**a**–**c**) and corresponding digital a.p. radiographs of the pelvis (**d-f**) in 3 different patients. A 28-year-old male (**a** and **d**) with hip dysplasia on both sides. Measurement of the right center-edge angle was almost identical with 16.6° for the virtual (**a**) and 16.7° for the digital radiograph (**d**). Note the sacrum and the sclerosis of the inferior pubic bones adjacent to the pubic symphysis (arrowheads) is better visible on the VR, because of bowel gas and soft tissue overlay on the DR. A 24-year-old female (**b** and **e**) with normal configuration of the left hip. The Sharp angle was within the normal range and was measured identical with 41.3° on VR (**b**) and DR (**e**). A 30-year-old female (**c** and **f**) with acetabular retroversion on both sides. VR (**c**) and DR (**f**) both show a crossover sign (arrows), posterior wall sign (the posterior acetabular wall is located medial to the center of the femoral head (asterisks)), and ischial spine sign (arrowheads) on both sides. DR, digital radiograph; VR, virtual radiograph

**Fig. 4:**
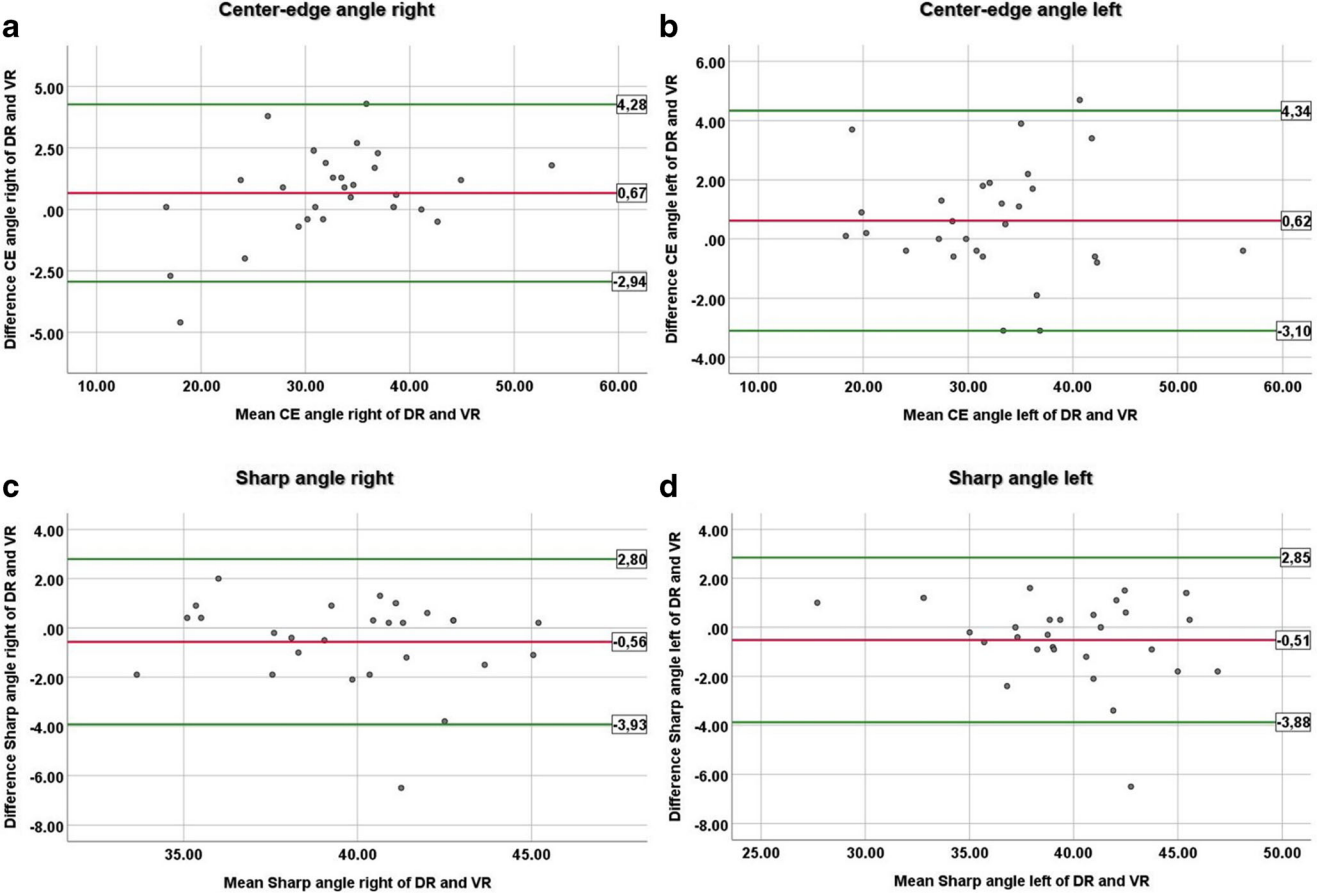
Bland-Altman plots for the right (**a**) and left (**b**) CE angle and the right (**c**) and left (**d**) Sharp angle. The upper limit, mean value, and lower limit are displayed as degrees in the boxes on the right of each plot. CE, center-edge; DR, digital radiograph; VR, virtual radiograph. Data are displayed in degrees

Inter-method reliabilities for measurement of CE and Sharp angles were excellent, as confirmed by the corresponding Bland-Altman plots (Figs. 3 and 4). For the right CE angle, the range between the lower limit and upper limit was 7.2°, for the left CE angle 7.4°, for the right Sharp angle 6.7°, and for the left Sharp angle 6.7°. Agreement between readers was good for the right Sharp angle on DR (ICC 0.87) and VR (ICC 0.9); for all other angle measurements, agreement was excellent (ICC 0.93–0.98).

## Discussion

Ultra-low-dose (ULD) CT with tin filtration achieved a 6.1-fold dose reduction compared to standard clinical CT of the pelvis, with a median effective dose of 0.38 mSv. ULD-CT with radiograph-equivalent dose showed clear depiction of bone anatomy and accurate detection of osseous pathologies, but with a markedly superior dose efficiency.

We were also able to calculate virtual pelvic radiographs (VR) of all patients based on ULD-CT data, which were equivalent to digital radiographs (DR).

A significant dose reduction with implementation of tin-filtered low-dose CT has been shown for the thorax [7–9] and the abdomen [10]. Braun et al [7] reported an 8.8-fold dose reduction for low-dose tin-filtered chest CT with a median effective dose of 0.24 mSv compared to 2.1 mSv for the standard protocol. For the tin-filtered low-dose protocol, they observed higher image noise and lower CNR, but a higher dose efficiency (FOM), which is consistent with our results. With a FOM of 927.6, the tin-filtered ultra-low-dose CT of the pelvis was also much more dose efficient than the standard CT (FOM 167.6; *p* < .001).

Messerli et al [8, 9] reduced the radiation dose of chest CT to the level of chest radiographs using the tin filter technique. Compared to the standard dose chest CT, a 92% dose reduction was achieved with a mean effective dose of 0.13 mSv for the tin-filtered ultra-low-dose CT. Similar sensitivities for computer-aided detection of solid pulmonary nodules were observed for both protocols [8]. In a different study, lung volumetry and quantification of emphysema were not different between the tin-filtered ultra-low-dose and the standard CT [9].

Leyendecker et al [10] reported a dose reduction of 81% for contrast-enhanced 100-kV abdominal CT using a tin filter protocol (mean effective dose 1.14 mSv) compared to mean 5.99 mSv for the standard protocol. The tin-filtered low-dose protocol in that study was also more dose efficient (FOM 10.6 vs. 2.49). Diagnostic confidence for abdominal pathologies was not different between protocols, which is consistent with our results. We also found that pelvic ULD-CT performs equally to standard CT in detection of fracture, osteoarthritis, cam deformity, and bone islands with no difference in diagnostic confidence. In their study with trauma patients, Hamard et al showed that ULD-CT was superior to radiographs in fracture detection, although standard CT technique was used. However, effective dose of the pelvic ULD-CT scans with mean 1.43 mSv was higher compared to our tin-filtered ULD-CT scans with median 0.38 mSv [22].

Calculation of virtual pelvic radiographs of ULD-CT data was successful for all patients. VRs were of good image quality and were equivalent to digital radiographs. Similar results were found by Sinatra and Moed [23], who did not find a difference in correct diagnosis of acetabular fractures between CT-generated and digital radiographs, although they used standard CT for this study. Bishop et al [24] compared digital radiographs of the injured pelvis and acetabulum to virtual radiographs that were created using volume rendering of standard CT data. Their results showed that virtual radiographs were superior to digital radiographs in visualization of acetabular fractures and visibility of anatomic landmarks of the acetabulum was superior as well. However, with the volume rendering approach to create virtual radiographs, they did not account for projection errors due to the parallel beam acquisition of the CT data, and therefore radiographic measurements (e.g., CE angle) cannot be accurately performed with their method.

In contrast, we calculated virtual radiographs with a cone beam–based projection algorithm to account for the difference in imaging technique, with parallel projection in computed tomography and cone beam projection in digital radiography. The settings of a digital orthopedic pelvic radiograph were closely simulated to generate virtual radiographs based on ULD-CT data. Furthermore, the ULD-CT was acquired in 15° internal rotation of the legs, according to the positioning in radiography [12].

With that approach, virtual radiographs showed correct anatomical projection compared to digital radiographs, which was confirmed by radiographic angle measurements.

With the tin-filtered ultra-low-dose CT in combination with virtual radiographs, we are able to lower the lifetime attributable risk of cancer of a pelvic CT examination to the level of digital radiographs, which was estimated by Wylie et al [2] for pre- and postoperative pelvic radiographs with 0.006% and 0.011% for 20-year-old male and female, respectively. According to their results, an additional preoperative standard pelvic CT with an effective dose of 5.06 mSv increased the risk to 0.055% and 0.094%, respectively, and the relative risk of lifetime cancer was 9.1 and 8.5 for males and females compared to digital radiographs alone. We are therefore convinced that the ultra-low-dose pelvic CT acquisition with tin filtration described in this manuscript has the potential to replace standard pelvic radiographs, as it produces both an accurate cross-sectional 3D data set and accurate virtual radiographs, but without the radiation dose of a standard CT. If necessary, the cone beam–based projection algorithm can also be adapted to produce other radiographic projections such as cross-table axial or obturator foramen views. Furthermore, assessment of small ossifications can also be performed on the calculated radiographs. The axial image reconstructions (1.5 mm) in soft tissue kernel of the ULD-CT allow for evaluation of the surrounding soft tissues (e.g., fatty muscle degeneration) despite higher image noise compared to standard CT. In the future, the use of machine learning might allow to generate CT images in diagnostic quality despite reducing the radiation dose of tin-filtered ultra-low-dose scans even below the level of digital radiographs. In a diagnostic accuracy study, Keller et al found no difference for torsion measurements of the lower limb between standard CT and simulated ULD-CT at dose levels as low as 1% of the original scan [25].

A limitation of our study was that patients who received the standard CT were scanned on 2 different CT machines for the clinical scans, with slightly different acquisition parameters. However, CT protocols were adjusted to equal dose parameters. Furthermore, for patients who did not receive the ULD-CT immediately after the standard CT on the same CT machine, scan length of standard CT and ULD-CT were not completely identical. Consequently, this potentially affected DLP and effective dose. In such cases, anatomical landmarks were used during ULD-CT acquisition to reduce the difference in scan length to a minimum.

In summary, tin-filtered ULD-CT of the osseous pelvis is feasible and showed a large dose reduction, equivalent to digital radiographs. ULD-CT showed clear depiction of bone anatomy and accurate detection of osseous pathologies. Furthermore, virtual pelvic radiographs of ULD-CT data can be calculated and are equivalent to digital radiographs.

## Supplementary information

ESM 1 (DOCX 659 kb)

## Abbreviations

CNR: Contrast-to-noise ratio
CTDI_vol_: Volume CT dose index
DLP: Dose length product
DR: Digital radiograph
FOM: Figure of merit
HU: Hounsfield unit
kV: Kilo volt
mAs: Milliampere seconds
mGy: Milligray
mSv: Millisievert
ROI: Region of interest
Sn: Tin filter
SNR: Signal-to-noise ratio
ULD-CT: Ultra-low-dose CT
VR: Virtual radiograph
VRT: Volume rendering technique

## Declarations

### Guarantor

The scientific guarantor of this publication is Reto Sutter, MD.

### Statistics and biometry

Tobias Götschi kindly provided statistical advice for this manuscript.

One of the authors has significant statistical expertise.

### Methodology

• prospective

• observational

• performed at one institution
