# Supplementary material for: Pelvic bone CT: can tin-filtered ultra-low-dose CT and virtual radiographs be used as alternative for standard CT and digital radiographs?
Source: Eur Radiol. 2021 Mar 12;31(9):6793–801. doi: 10.1007/s00330-021-07824-x (PMC8379132; doi:10.1007/s00330-021-07824-x)

## Supplementary material

# Materials and methods

## Pelvic phantom

## A standard pelvic phantom was used for several consecutive CT scans with a range of different acquisition parameters, up to ultra-low-dose levels. First the phantom was scanned with the standard CT protocol on a 128-slice CT scanner (SOMATOM Edge Plus, Siemens Healthineers) with automated tube voltage selection (CARE kV, reference 120kV) and tube current modulation (CARE Dose4D, reference 147mAs), a collimation width of 0.6 mm, a rotation time of 0.5 s and a pitch of 0.8. Afterwards successive tin-filtered CT scans with 100kV and 140kV were performed with a fixed tube current (Sn100kV: 500 – 25mAs, Sn140kV: 200-25mAs) on the same CT scanner. Further settings were: a collimation width of 0.6 mm, rotation time of 1 s and a pitch of 0.8. CT parameters were extracted from the dose report and effective dose was estimated by multiplying the DLP with a standard conversion factor *k* for the adult pelvis of 0.013 mSv/mGy*cm [13].

## Pelvic cadavers

3 cadavers with an intact pelvis were used for the second part of optimization of CT parameters and to establish a clinical protocol for an ultra-low-dose CT scan of the osseous pelvis with tin filtration. All cadavers were free of metal implants or tumor. Each cadaver was scanned with the standard CT protocol followed by successive tin-filtered ultra-low-dose CT scans with Sn100kV and Sn140kV up to a radiation dose of pelvic radiographs. CT parameters of the Sn100kV scans were adjusted to the parameters of the Sn140kV scans to achieve dose-equivalent protocols for comparability of image quality (Sn 100kV: 138-277mAs, Sn140kV: 25-50mAs). All standard CT and ULD-CT scans were performed on the same CT scanner as the phantom scans with the same settings for collimation width, rotation time and pitch. The standard conversion factor *k* for the adult pelvis of 0.013 mSv/mGy*cm [13] was used and multiplied with the DLP to estimated effective dose of each CT scan. 12 dose-equivalent ULD-CT scans (6 scans with Sn100kV and 6 scans with Sn140kV) of each cadaver were rated blinded and independently by two musculoskeletal radiologists (C.S. and J.G., both with 7 years of experience) on a 4-point Likert scale: depiction of anatomy (1=poor, 2=fair, 3=moderate, 4=good), image noise (1=very high, 2=high, 3=moderate, 4=minimal) and image artifacts (1=very strong, 2=strong, 3=weak, 4=none) (supplementary table 1).

For all CT acquisitions the scan region included the superior iliac crest to the inferior lesser trochanter of the femur. All CT and ULD-CT scans were reconstructed with advanced modeled iterative reconstruction (ADMIRE) strength level 3 in the axial (3 mm), coronal (2 mm) and sagittal (2 mm) image plane using a bone kernel (Br 57) and in the axial (1.5 mm) image plane using a soft tissue kernel (Br 38).

**Results**

CT parameters of the phantom scans up to the dose of digital pelvic radiographs (estimated with mean 0.37mSv (± SD 0.14mSv)) were 300mAs for the Sn100kV (0.34mSv) and 55mAs for the Sn140kV (0.34mSv) protocol.

For the 3 cadavers estimated effective dose was mean 3.08mSv (± SD 1.51mSv) for the standard CT protocol and mean 0.36mSv (± SD 0.05mSv) for the Sn140kV/50mAs and the dose-equivalent Sn100kV/277mAs ultra-low-dose CT protocol, resulting in 88% dose reduction. Both readers rated the protocol Sn140kV/50mAs as the best protocol (supplementary table 3), (supplementary Fig. 1).

**Supplementary figure legends**

**Supplementary Fig. 1** A 73-year-old male cadaver scanned with the standard (A) and ultra-low-dose protocol with tin filtration (Sn140kV/50mAs; C). Note bone island in the left ischial bone (arrow). A 84-year-old male cadaver scanned with the standard (B) and ultra-low-dose protocol with tin filtration (Sn140kV/50mAs; D). Note partial ankylosis of the right and left sacroiliac joint (arrows) and arteriosclerosis (arrowheads).


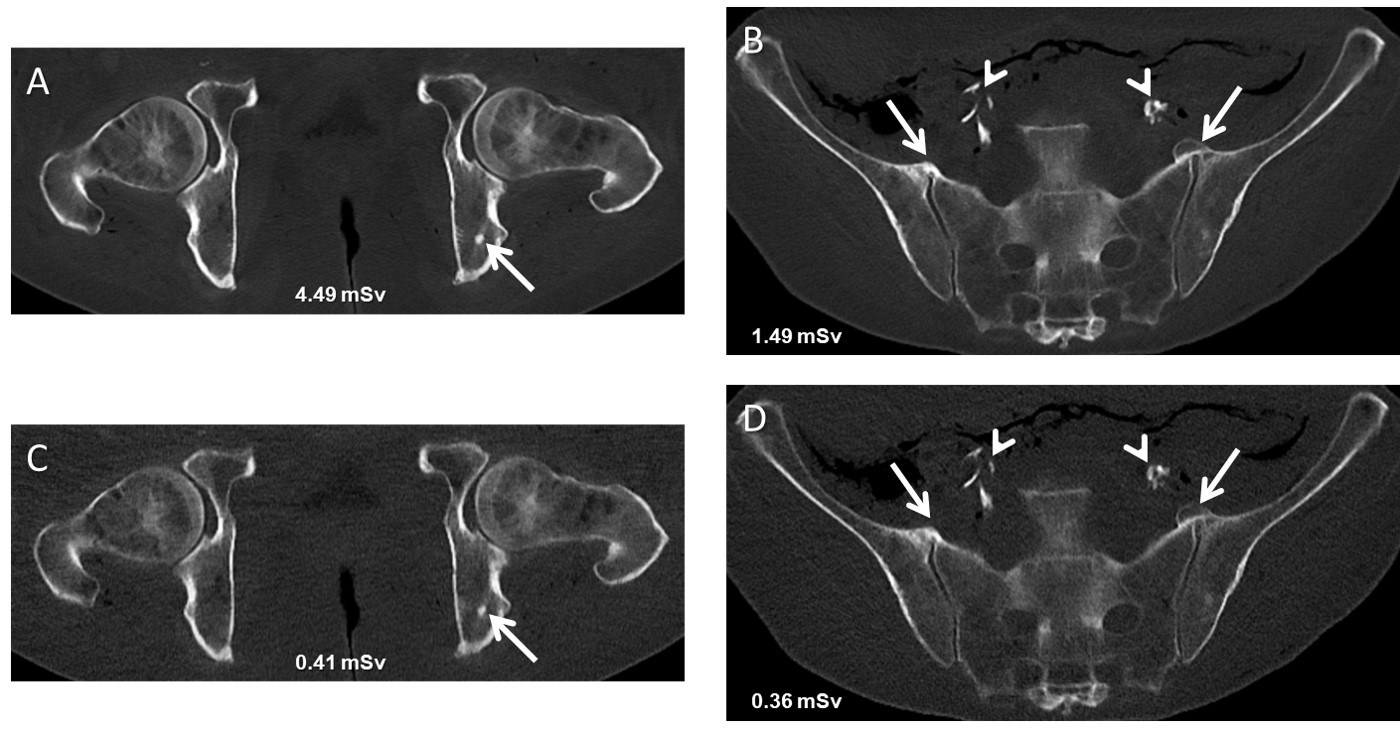


**Supplementary Fig. 2** A 29-year-old female with acetabular retroversion of the right hip. Reformatted axial CT image of the right proximal femur at the level of the lesser trochanter, scanned with the standard protocol (110kV/120mAs; A) and ultra-low-dose protocol with tin filtration (Sn140kV/50mAs; B) show scattering (arrows) and beam hardening (arrowheads) on the standard CT image (A) while these artifacts are not visible on the ultra-low-dose CT image (B).


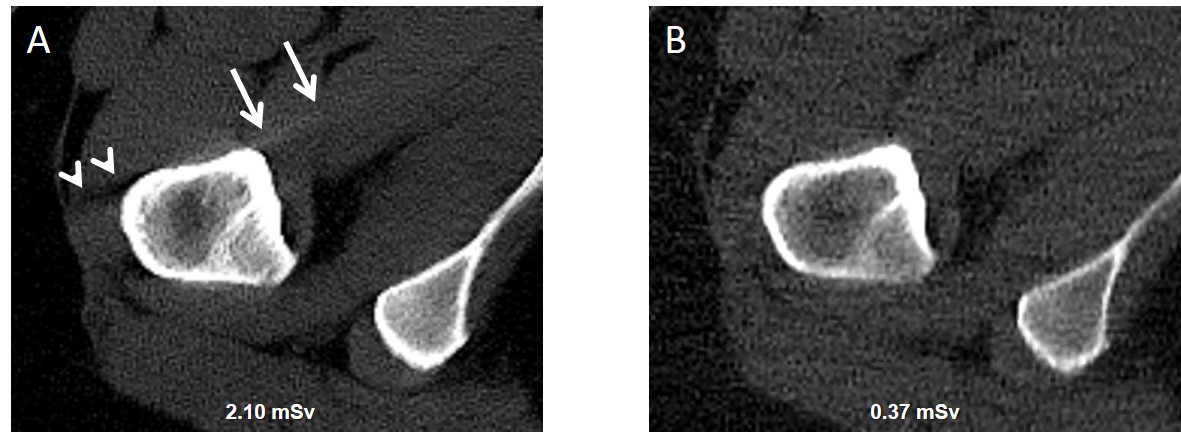


**Supplementary table legends**

**
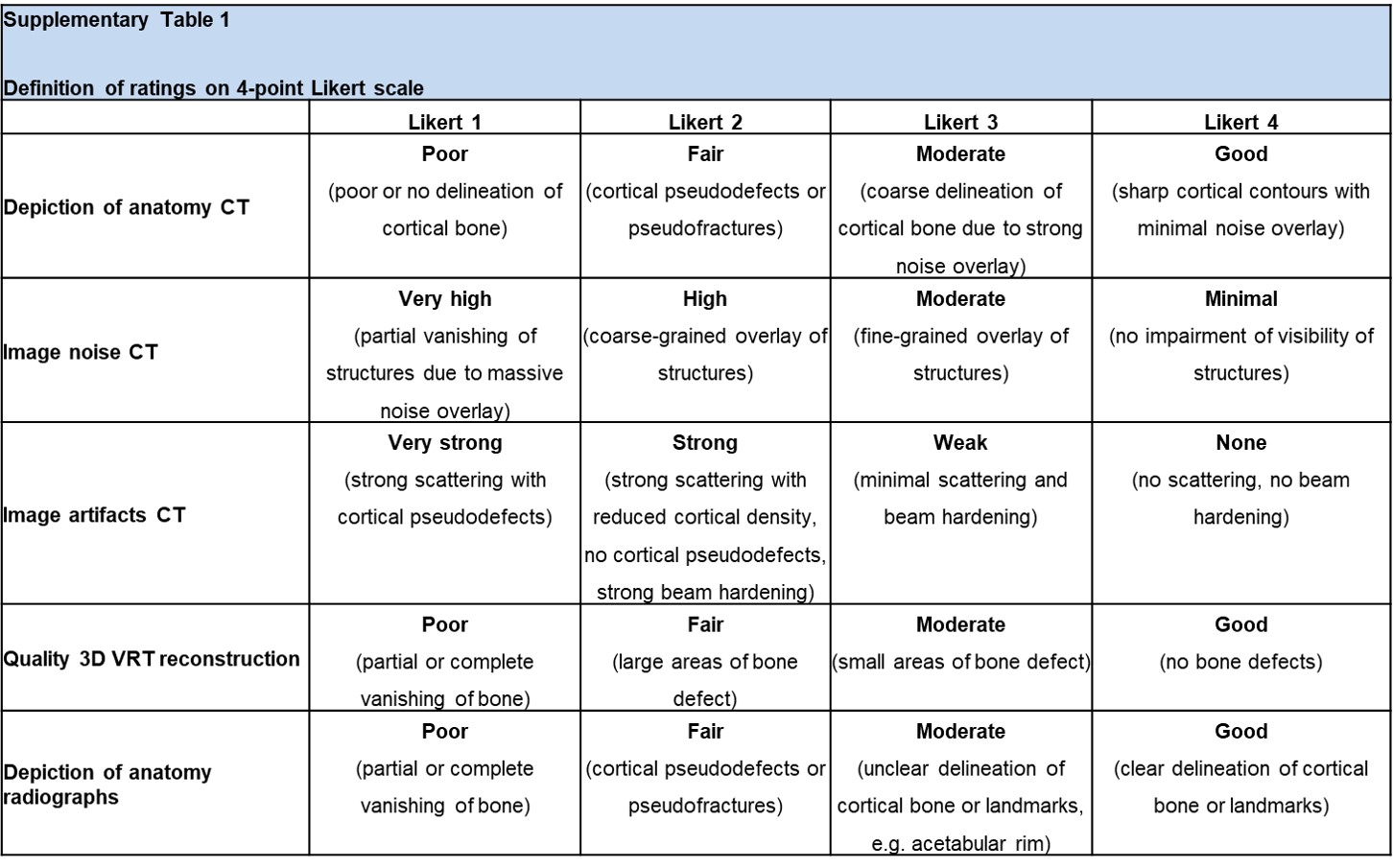
**

**Supplementary table 2**

*Abbreviations:* DR = digital radiograph, CT = standard CT, NA = not applicable, ULD-CT = ultra-low-dose CT, VR = virtual radiograph


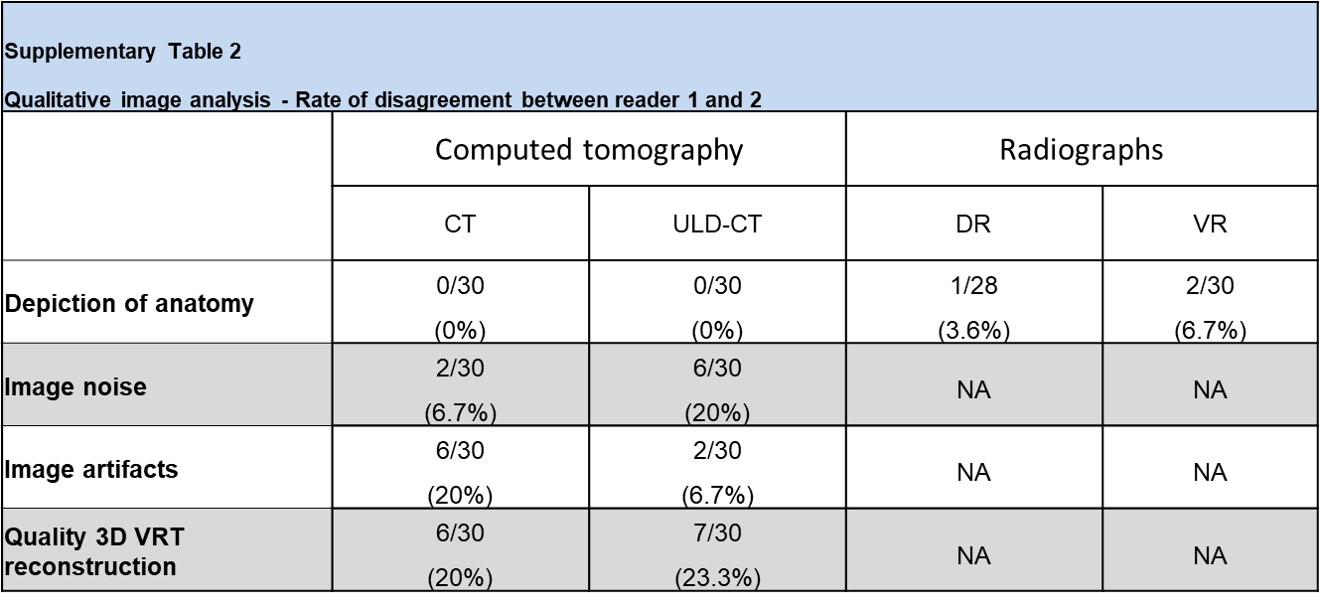


**Supplementary table 3**

*Abbreviations:* Sn = tin filter


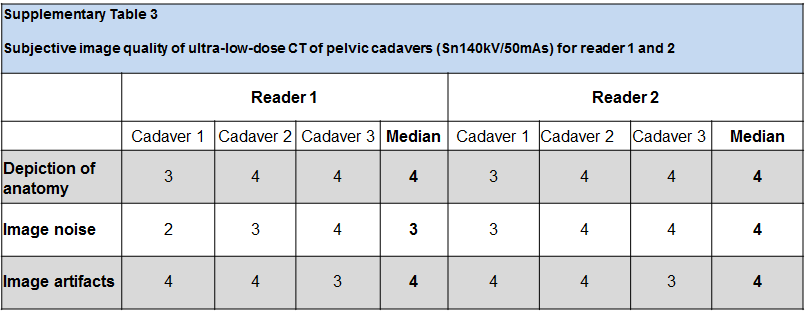

Supplement: Supplementary file 1 — (DOCX 659 kb) [file 330_2021_7824_MOESM1_ESM.docx]
